# The Development and Psychometric Assessment of Chinese Medication Literacy Scale for Hypertensive Patients (C-MLSHP)

**DOI:** 10.3389/fphar.2020.00490

**Published:** 2020-04-30

**Authors:** Zhuqing Zhong, Shuangjiao Shi, Yinglong Duan, Zhiying Shen, Feng Zheng, Siqing Ding, Aijing Luo

**Affiliations:** ^1^Nursing Department, Third Xiangya Hospital, Central South University, Changsha, China; ^2^Xiangya Nursing School, Central South University, Changsha, China; ^3^Department of Cardiology and Cardiovascular, Third Xiangya Hospital, Central South University, Changsha, China; ^4^Key Laboratory of Medical Informatics Research, Central South University, College of Hunan Province, Changsha, China

**Keywords:** hypertension, medication literacy, scale, reliability, validity

## Abstract

**Objective:**

To develop the medication literacy scale for patients with hypertension, and to test the reliability and validity of the scale.

**Methods:**

The initial draft of the scale was formulated based on the operationalization of medication literacy with four core elements of knowledge, attitude, skill, and practice, and was developed through procedures of literature review, interviews to hypertensive patients, and research group discussion. Expert panel meeting, interviews, and pre-test on the initial draft of the scale to 10 hypertensive patients, as well as a two iterations of expert feedback were used to form a primary medication literacy scale for pilot investigation and item selection. In this study, 260 patients with hypertension in Changsha city of China were purposively selected to conduct a pilot survey using the primary medication literacy scale. After item selection by a series of statistical analysis method and item re-wording according to patients’ feedback, the scale was revised to form a formal investigation scale with four domains and 37 items. A formal investigation was carried out on 650 patients with hypertension selected purposively in a tertiary general hospital and two community health service centers in Changsha city of China. The reliability and validity of the scale were analyzed.

**Results:**

Finally, the formal scale consists of four domains on knowledge, attitude, practice and skills, 11 sub-factors and 37 items in total. The scale-level content validity index (S-CVI/Ave) of this scale was 0.968, and the I-CVI for each item ranged from 0.833 to 1.000, indicating a good and acceptable content and face validity. The Cronbach’s α coefficient was 0.849 for the overall scale and ranged from 0.744 to 0.783 for domains. The Pearson’s correlation coefficients between domains and the total scale were ranging from 0.530 to 0.799. Besides, the Pearson’s correlation coefficient among domains of the scale ranged from 0.157 to 0.439. The Spearman-Brown split-half reliability coefficient was 0.893 for the total scale and ranged from 0.793 to 0.872 for domains. The test-retest reliability coefficient of the total scale was 0.968 and ranged from 0.880 to 0.959 for domains. Four domains of knowledge, attitude, skill, and practice were identified through the exploratory factor analysis and confirmatory factor analysis from each domain. The total explained variation of domains for the overall scale was 51.420%. Eleven sub-factors for domains were extracted through respective exploratory factor analysis from each domain, and the total explained variation of sub-factors for its belonging domain were ranging from 56.111 to 64.419%. The confirmatory factor analysis showed the fit indices of the four-domain model were as follows (χ^2^/df=2.629, GFI=0.804, AGFI=0.777, RMR=0.012, IFI=0.746, RMSEA=0.066, PNFI=0.599, PCFI=0.689), which indicated an acceptable model fit.

**Conclusions:**

The medication literacy scale for hypertensive patients has good reliability and acceptable validity, which is suitable and acceptable for evaluating the medication literacy level of hypertension patients in China. In the future, further construct and model fit validation and English translation with appropriate adaptation of this whole scale are required, so that this scale can be further validated and applied worldwide.

## Introduction

Medication safety problem has always been the focus of healthcare providers and public health community scholars. Researches across the globe reported that there were certain safety problems in medication taking process for hypertensive patients (Liu et al., 2016; Rahmawati and Bajorek, 2017). This present study was referred to and complied with Pouliot’s study on conceptualization of medication literacy. Medication literacy is the degree to which individuals can obtain, comprehend, communicate, calculate, and process patient-specific information about their medications to make informed medication and health decisions in order to safely and effectively use their medications, regardless of the mode by which the content is delivered (e.g., written, oral, and visual) (Pouliot et al., 2018). Compared with the concept of pharmacotherapy literacy studied by King et al. (2011), international consensus on the concept of medication literacy was patient-specific and focused on the individual’s ability to use medication correctly and safely. It was the first definition that took into consideration the context of the individual (Pouliot et al., 2018). In Pouliot’s study, patient’s beliefs and personal circumstances were considered into the medication literacy conceptualization, it was a two-way dialogue between patients and their healthcare providers about the benefits, risks, and alternatives of treatment, in order to achieve optimal goal and outcomes of individual’s medication literacy (Pouliot et al., 2018). Specifically, for hypertensive patients, their beliefs and attitudes to the severity of hypertension disease, to the necessity of antihypertensive taking and of the treatment plan discussion with healthcare providers, as well as their self-efficacy were playing a critical role in their adherence to antihypertensive drug taking and self-management (Al-Noumani et al, 2018; Al-Noumani et al, 2019; Qvarnström et al, 2019). Therefore, hypertensive patients’ attitudes, including their beliefs related to hypertension severity and susceptibility, their beliefs to the effectiveness or necessity of taking antihypertensive medication, as well as their self-efficacy in hypertension disease management, were incorporated into the conceptualization and operationalization of hypertensive patients’ medication literacy.

In Pouliot’s study, an internationally consensus on the concept of medication literacy was achieved, and it was divided into four clusters representing: 1) type of information necessary for optimal and safe use of medication, 2) skills and abilities, 3) format of information, and 4) the outcomes and goals of medication literacy. In this four elements, type and format of information that is necessary for optimal and safe medication use was emphasized. The medication related information is always appearing on doctor’s prescription sheets or on medication instruction labels inserted in the pill boxes in China. Therefore, it is very important that patients can read, understand and calculate according to the information on the medication instruction labels and prescription sheets. It is feasible and applicable to involve print information for medicines in assessing patients’ skills of using medication and it is also very important. Furthermore, patients’ skills, e.g., how to interpret medication labels such as dosing and measurements was also emphasized in the development of medication literacy measure (Sauceda et al., 2012). In addition, King, Ubavić et al. and Krajnović et al. referred to four domains of pharmacotherapy literacy: knowledge, understanding for health information (written and spoken), numeracy, and access to medicine-related information (King et al. 2011; Ubavić et al., 2018a; Ubavić et al., 2018b; Krajnovic, 2019). The understanding for written or spoken information and numeracy were also emphasized in the concept of pharmacotherapy literacy. Obviously, for patients, understanding medication information sheets or labels involving dosing or frequency and necessary numerical skills were pivotal to perform correctly in the pharmacotherapy process on their own. Yeh *et al*. developed the first Chinese medication literacy measure for general population mainly based on evaluating patients’ abilities and skills to interpret medication information sheets or instruction labels of prescription or non-prescription medicines (Yeh et al., 2017). Medication Literacy Assessment Questionnaire with 30 items in five categories developed and validated by Horvat *et al*. also focused on assessing patients’ abilities to understand medication instruction sheets and calculate (Horvat et al., 2017). Therefore, for hypertensive patients, the abilities and skills to use medication optimally and safely according to printed and limited medication information are one of the elements we considered to operationalize the concept of medication literacy for hypertensive patients in the present study. Specifically, skills related to comprehending medication information labels and doing calculations, including finding the correct information about the drug’s indications, warnings, dosing directions, expiration date, side-effects, and calculating the drug’s doses, frequency, and next dosing time, were incorporated in the operationalization of medication literacy for hypertensive patients.

Knowledge for medication specific for patients with certain kinds of disease was also incorporated in the concept of pharmacotherapy literacy and medication literacy (King et al., 2011; Sauceda et al., 2012; Ubavić et al, 2018a; Ubavić et al, 2018b; Krajnović et al., 2019). Therefore, knowledge for hypertension disease, treatment principal, and antihypertensives taking has been applied into operationalizing medication literacy for hypertensive patients in this present study.

One of the clusters representing identified in the international consensus study of the concept of medication literacy is the outcomes and goals of medication literacy (Pouliot et al., 2018). Optimal outcomes and goals in the process of medication use was expected to be achieved. For one, patients’ informed decisions on processing medication in a correct way were generated from their knowledge and attitudes to specific disease and medication, as well as their abilities to interpret and calculate according to medication instructions or doctors’ prescriptions. For another, patients’ practice on correct medication use was definitely driven by their own decisions. Therefore, practice on correct medication use is one of the very indicators of optimal outcomes and goals in the process of medication use. In addition, parental practice on medication use was associated with their pharmacotherapy literacy (knowledge, understanding, numeracy, and access to medicine-related information) (Krajnović et al., 2019). Therefore, practices related to adherent medication taking behavior specific for hypertensive patients, their decision making behavior for choosing medication, medication information-seeking, and dissemination behavior, as well as adverse effects surveillance and blood pressure self-monitoring behavior were incorporated as one of the operationalized elements in medication literacy specific for hypertensive patients of the present study. According to knowledge-attitude-practice model (KAP) (Alzghoul and Chew Abdullah, 2015), it is confirmed that knowledge and attitude contributes to correspondingly informed practices. Therefore, in this study, the identified four operationalized elements of knowledge, attitude, skills, and practice of medication literacy for hypertensive patients could be consistent with this well-known KAP model with skills added into its functioning procedure in the context of medication use.

Safe and correct self-medication was a leading contributor to the optimal blood pressure control for hypertensive patients (Hu, 2010). For hypertensive patients, adherence to prescribed medication regimen and taking antihypertensives in a correct and safe way are prerequisites for achieving optimal blood pressure control and improving their long-term challenging progressive disease state. Therefore, the correct medication use and the effectiveness of medication therapy for hypertensive patients might mainly depend on their understanding of related knowledge about medication, attitudes to antihypertensive medication taking, skills on how they should administer the prescribed medication in a right way, adherent medication taking behavior and related practices on appropriately dealing with adverse reaction and blood pressure monitoring (Shi et al., 2019). Therefore, it is of great significance to assess the level of medication literacy of hypertensive patients, which could be a first pivotal step to target gaps and patients’ problems of pharmacotherapy. Then targeted and tailored counseling and interventions to prompt persistent, correct, and safe antihypertensive therapy for patients could be implemented.

However, there is a dearth of specific medication literacy scale for hypertensive patients currently, although several medication literacy measurements for general population have been found (Sauceda et al., 2012; Yeh et al., 2017; Horvat et al., 2017). Pharmacotherapy literacy questionnaire for parents of pre-school children and medication health literacy screen measure were also found (Stilley et al., 2014; Ubavić et al., 2018b).

Hence, based on previous literature research and operationalized elements of medication literacy, a specific assessment scale of medication literacy for Chinese hypertensive patients has been developed in the present study, and four domains of knowledge, attitude, skill, and practice were included, the reliability and validity test were also performed.

## Methods

### Phases of Development

An initial draft of the scale (52 items) was developed through literature review, hypertensive patient interview, and house group discussion in research group. Subsequently, the initial drafted item pool was assessed in an expert panel meeting, applied in a pre-test performed to 10 hypertensive patients and the feedback was recorded to remove or revise items. Therefore, a primary medication literacy scale with 41 items was reached. In addition, another two items were excluded according to feedbacks and ratings of a two-round expert consultation. Finally, the 39-item primary scale was used to conduct a pilot study with 260 hypertensive patients. After item selection with several statistical analysis, an ultimate medication literacy scale with 4 domains and 37 items was completed. The flow chart of the scale development procedure and the detailed final 37-item scale has been attached as **Appendices** in the **Supplementary Material**.

### Initial Item Pool Establishment

Knowledge-Attitude-Practice model (Alzghoul and Chew Abdullah, 2015), health belief model (Peng et al., 2014), plan behavior theory (Cheng et al., 2012), and health literacy (Sorensen et al., 2012) were analyzed and incorporated, along with analysis on operationalization of “medication literacy” throughout related literature review (King et al., 2011; Pouliot et al., 2018). In addition, patients’ skills to use medication correctly and safely were also incorporated into the conceptualization of medication literacy according to literature analysis and health literacy framework (Sorensen et al., 2012). Methods of derivation, synthesis, and theory analysis developed by Walker (Butcher, 2006; Walker and Avant, 2010) were used for concept establishment. Finally, medication literacy was conceptualized and operationalized.

Based on the concept of medication literacy and its four core elements, the operationalized framework of medication literacy for hypertensive patients with four domains of knowledge, attitude, skill, and practice was identified. Firstly, we established an indicator system of medication literacy for hypertensive patients based on the operationalized four core elements. Then the initial item pool of this scale was developed based on this indicator system accordingly.

Methods for development of initial item pool:

- Related literature review of existing researches about instruments for assessing medication literacy level of general population or some other disease-specific population, some items were extracted and adapted from these existing medication literacy questionnaires or scales. Some of the items were formulated referring to existing measurements for assessing hypertension treatment adherence and to those studies on medication use for hypertensive patients.
- The results of interviews on hypertensive patients’ antihypertensive medication taking and hypertension disease management. All items were developed based on the four core elements of hypertensive patients’ medication literacy of knowledge, attitude, skill, and practice.
- Then, after research group discussion, an initial draft of the scale with 52 items was reached through the above procedures.

### Primary Medication Literacy Scale Development for Pilot Investigation

- An expert panel meeting was convened, related experts specialized in cardiovascular research and pharmaceutical research were invited to examine the clarity of drafted items as well as each item’s relevance and appropriateness to its belonging construct. Some inappropriate items were removed or revised and some highly relevant extra items suggested by experts were supplemented;
- Interview and pre-test of the initial drafted scale to 10 hypertensive patients: after items were revised according to the advises in expert panel meeting, the revised initial drafted scale from above were applied to the interviews performed to 10 recruited hypertensive patients. The questions as well as suggestions put forward by interviewed patients on each item of the revised initial drafted scale were recorded, according to which complex items with technical words that were hard to understand by patients were revised;
- Focus group discussion: the advices of expert panel meeting and the results of pre-test interviews for hypertensive patients were integrated and synthesized through discussion by research group, resulting in a 41-item scale after appropriate revisions to items and 11 items removed. Furthermore, there were another two items being removed according to suggestions generated from a two-round expert consultation. Therefore, a primary medication literacy scale for hypertensive patients involving 39 items was accomplished.

### Content and Face Validity

Six experts have been also invited to participate in a two-round expert consultation to appraise on the construct and items of the primary medication literacy scale with 39 items in this study. Based on every expert’s understanding of the definition and connotation of hypertensive patients’ medication literacy, as well as its four core elements, constructive amendments, and item suggestions were required to be given. Therefore, supplements, expurgations, and revisions to some items or contents have been made accordingly.

Inclusion criteria for experts: a. with over 10 years of work experience in the cardiovascular department; b. with doctoral degree or above; c. at least with professional title of associate professor or with deputy director or above; d. experts who master in the development and psychometric assessment of a scale; e. experts who were interested in this research and willing to offer advices or suggestions. Finally, two clinical pediatric professionals, two nursing professionals specialized in hypertension management and psychometric assessment on scale, and two pediatric pharmaceutical professionals were involved.

The authority coefficient of each expert has been calculated in a comprehensive way, including appraises on experts’ level of academic ability, judgmental reference to suggestions generated on items of the scale, and their familiarity degree to the concept of medication literacy. The experts were asked to rate each item’s importance and appropriateness/relevance on a four-point scale, ranging from “high relevance” to “irrelevance.” The intended meaning and clarity of the items were checked. High relevance scored four indicating strong correlation and high relevance between the item and its belonging domain and the overall scale. Relevance scored three indicating certain correlation between the item and its belonging domain and the overall scale. Slight relevance scored two identifying weak correlation, and irrelevance scored one indicating there was no correlation between the item and its belonging domain and the overall scale. In addition, the experts were encouraged to modify and/or comment on the items and explain their rationales. Two iterations of feedback and discussion among the experts generated the revised version. Content and face validity for the scale was assessed by content validity index (CVI) (Martuza, 1977; Hambleton et al., 1978). Therefore, the content validity index for the overall scale (S-CVI/Ave) and for each item of this scale (I-CVI) were calculated. After random consistency was calibrated by applying with Kappa value (K^*^) (Polit et al., 2007), items with I-CVI (CVI in item level) < 0.78 were excluded (Lynn, 1986; Shi et al., 2012). Significant items were retained whereas non-significant items were excluded. The final content validity index for this scale was calculated according to experts’ ratings for each remaining items in the second iteration. The face and content validity were established at this point.

### Pilot Survey

Purposive sampling method was applied, and a total of 260 hypertensive patients collected from a tertiary hospital and a community health service center in Changsha city of China were participated in this pilot survey. The primary medication literacy scale with 39 items was used as investigation tool. Inclusion criteria were as follows: Patients who a. were diagnosed as hypertension according to the 2016 revised version of guidance for hypertension prevention and treatment in China, which is systolic BP>=140 mmHg or diastolic BP>=90 mmHg; b. have been on antihypertensive treatment and taking antihypertensives for at least 2 weeks, these included both those newly diagnosed and being treated with antihypertensive medication for a short period of time and those who were already on antihypertensive medication treatment for a longer period of time; c. aged over 18 years old; d. can communicate with others and have the ability of reading and comprehension; e. were willing to participate in this study and signed the consent forms; exclusion criteria were as follows: patients who a. were diagnosed as psychologically and mentally ill by ICD or have been on a mental pharmacotherapy; b. have severe or acute hypertension or other uncontrolled cardiovascular and cerebrovascular diseases such as heart failure in New York Heart Association Class III or IV, or unstable angina; c. have dementia or cognitive impairment, severe diseases of other organs or systems, such as cancer; d. were with hearing and communication disability.

In the process of the investigation, the problems of item understanding and wording as well as construct of the primary assessment scale were checked again, and questions about the clarity and accuracy of the expression of each item were recorded.

Meanwhile, collected data were statistically analyzed using IBM SPSS 25.0 for item selection. After item selection based on the results of a series of statistical analysis, a complete and final medication literacy scale can be developed. During the period of questionnaire investigation, participants’ timely feedback on items or the whole questionnaire was focused on and improvements were made. Items that were questionable or confusing for participants were given appropriate revision.

In this pilot survey, out of the 260 distributed questionnaires, a total of 252 completed questionnaires were collected back and checked validity. The response rate was 96.60%. Statistical analysis methods for item selection were used. Item discrimination of t-test, Cronbach’s alpha (α) were calculated to remove or revise items.

Methods for item selection:

- Item discrimination analysis: total scores of collected questionnaires were listed in sequence of numeric value from high to low, among which 27% of the collected questionnaires with high total scores from the highest one were defined as high score group, 27% of the collected questionnaires with low total scores from the lowest one were defined as low score group, then scores of each item in two groups was tested difference using independent t-test method. Items with scores that have no significant difference between in high score group and in low score group were excluded.
- Correlation coefficient method: the Pearson’s correlation coefficients were calculated, including those between each item and its belonging domain, between each item and the overall scale, as well as the Pearson’s correlation coefficient between each domain and the whole scale. Items with Pearson’s correlation coefficient *r* < 0.3 were removed (P < 0.05). Considering the specialty practicalities, appropriate item remove and revisions were made.

### Formal Investigation

In formal investigation stage of this research, purposive sampling method was used. Four hundred hypertensive patients were collected from inpatient and outpatient departments of a tertiary hospital in the city of Changsha, China, 250 hypertensive patients were collected from two community health service centers in Changsha city of China from April to June, 2016. Therefore, a total of 650 eligible hypertensive patients participated in this investigation. Out of the 650 distributed questionnaires, 637 were collected back and checked completion and validity. The response rate was 98.00%. Among these participants, they aged from 18 to 90 years old, and the average age of them was (57.49 ± 15.12) years; in addition, 336 out of 637 participants were male (52.7%), 542 has been married (85.0%), 149 participants had an education level of primary school or below(23.4%); 462 participants were employed (72.5%); 220 (34.5%) participants have been diagnosed as hypertension for more than 10 years; 421 (66.1%) participants had a family history of hypertension (**Table 1**). This study involving human participants was reviewed and approved by the Ethics Committee of the Third Xiangya Hospital. The participants have all provided their written informed consent to participate in this study.

**Table 1:** Patient characteristics (n=637).

Items	Group	N	%
Age (years)*	18~45	131	20.6
	46~60	183	28.7
	61~90	323	50.7
Gender	Male	336	52.7
	Female	301	47.3
Education level	Primary and below	149	23.4
	Junior middle school	158	24.8
	High school	115	18.1
	Junior College	81	12.7
	College degree and above	134	21.0
Annual household income Chinese CNY (¥)	<10,000/year.	112	17.6
	10,000~29,999/year	131	20.6
	30,000~49,999/year	171	26.8
	50,000~99,999/year	101	15.9
	≧100,000/year	122	19.2
Marital status	Married	542	85.0
	Unmarried	35	5.5
	Divorced or widowed	60	9.5
Occupational status	Employed	462	72.5
	Retired	133	20.9
	Unemployed	42	6.6
Registered residence	Urban	380	59.7
	Countryside	257	40.3
Duration of hypertension	<3 years	187	29.4
	3-years	82	12.9
	5-years	146	22.9
	≧10 years	220	34.5
Family history of hypertension	Yes	421	66.1
	No	216	33.9

*The mean for age was 57.49 years with a standard deviation of 15.12.

#### Validity Test

Content validity and construct validity were checked and tested. Content validity was assessed by calculating the content validity index of each item (I-CVI) and the content validity index of the whole scale (S-CVI/Ave) (Martuza, 1977; Hambleton et al., 1978). The calculations have been figured out according to the feedbacks of the second iteration expert consultation. I-CVI≥0.78 indicates good item-level content validity (Lynn, 1986). S-CVI/Ave ≥ 0.90 means a good scale-level content validity (Waltz et al., 2005). In this stage, we also calculated the adjusted kappa value (K*) to calibrate the chance agreement degree of experts’ appraise and ratings on each item. K* ranges from 0.40 to 0.59 indicating a grudgingly acceptable degree of chance agreement, 0.60–0.74 indicating an acceptable degree of chance agreement, K* > 0.74 means a good result of calibrating the degree of chance agreement (Polit et al., 2007). In addition, the authority coefficient of each expert in this expert panel were calculated based on experts’ characteristics.

Construct validity of this scale was assessed by conducting exploratory factor analysis to identify its constructs and to assess whether data was grouped as anticipated. Confirmatory factor analysis was conducted to confirm the identified constructs and principal components with related indices.

Out of the 637 collected responses, we used 257 questionnaires to explore factor structure of this scale, and the rest 380 instruments of the data were used to confirm factor structure with fit indices. Absolute fit indices namely Chi-square value/freedom degree (χ^2^/df), the goodness-of-fit (GFI), absolute goodness-of-fit (AGFI), root-mean-square error of approximation (RMSEA), and standardized root mean square residual (SRMR) were calculated. In addition, incremental fit indices (IFI), parsimony fit index including parsimony normed fit index (PNFI), parsimony comparative fit index (PCFI) were also noted (Wu, 2009). A good model fit was highlighted if these indices reached values as follows (Joöreskog and Long, 1993): GFI, AGFI, and IFI were > 0.90, RMR < 0.05, indicating good model fit (Pett et al., 2003; Hair et al., 2009; Shima et al., 2015). For RMSEA, the value ranges from 0.05 to 0.08 suggesting acceptable model fit, the value less than 0.05 shows great model fit. Generally, RMSEA, SRMR values < 0.08 indicate acceptable model fit (Pett et al., 2003; Sun, 2005; Hair et al., 2009; Shima et al., 2015). A value for parsimony fit index (PNFI, PCFI) > 0.5 was considered satisfactory (Mulaik et al., 1989). χ^2^/df was an absolute fit index. For the χ^2^/df, the smaller of the value, the better of the model fit, and χ^2^/df < 3 indicates a good model fit (Hu and Bentler, 1998). Structure equation modeling was carried out using IBM SPSS AMOS version 25.

Convergent validity was assessed by calculating average factor loading of each construct. The convergent validity for the scale was satisfied if the standardized factor loadings of this scale were greater than 0.5 (Bagozzi et al., 1981; Hair et al., 2009; Hair et al., 2010). Discriminant validity was also identified by calculating the average variance and squared correlation coefficient among domains. The scale had an acceptable discriminant validity if the average variance was greater than squared correlation coefficients (Streiner and Norman, 1995; Bowling, 2009; Hair et al., 2010).

#### Reliability Test

Internal consistency was assessed using Cronbach’s alpha (α) values. A value of at least 0.7 was considered satisfactory internal reliability (Nunnally and Bernstein, 1994; Sushil and Verma, 2010). Spearman-Brown split-half reliability was also calculated, a correlation coefficient ≥ 0.7 indicates good internal reliability (Cronbach, 1951; Karahan Okuroglu et al., 2019). The test-retest reliability was measured by Pearson’s correlation coefficient between two time-points with a gap of 2 weeks in 40 randomly collected hypertensive patients. The value of correlation coefficient over time more than 0.75 (P < 0.05) was considered good test-retest reliability (Lahey et al., 1983; Cohen, 1988; De Vellis, 1991).

#### Scoring Criteria for Chinese Medication Literacy Scale for Hypertensive Patients

This research scale measured medication literacy level of hypertensive patients across four domains namely knowledge about hypertension disease, treatment, and antihypertensive medication, attitude, skill, and practice for medication administration. For items in domains of knowledge and skill, answering right for each item scores 1, and answering wrong or answered “I don’t know” scores 0. A response option of 5-point Likert scale for each item in attitude domain was used (totally agree, agree, not sure, disagree, totally disagree), in which scores of 1.0, 0.75, 0.5, 0.25, 0 were applied accordingly. In the practice domain, a response option of 5-point Likert scale was also applied for each item (always, often, sometimes, seldom, never), and scores of 1.0, 0.75, 0.5, 0.25, 0 were also used accordingly. In addition, there were five items in the attitude domain and one item in practice domain scoring reversely. The summed total score on this 37-item scale ranged from 0 to 37, with higher scores indicating higher medication literacy level.

## Results

### Scale Construct and Items Generation

An initial entry pool of 52 items was established in this study at the beginning. Then, a scale with 41 items was developed after 11 items were excluded through expert panel meeting, interviews, and pre-test to several patients as well as the focus group discussion. Subsequently, the primary medication literacy scale for hypertensive patients with 39 items for pilot survey has been formed after 2 items being excluded according to suggestions generated from the two-round expert consultation. Finally, two items with low discrimination were excluded after analysis of item discrimination and correlation coefficient method on the collected data from pilot survey. (Item A4: I am more willing to try traditional Chinese prescription; item A10: I worry about the side effects of long-term antihypertensive treatment). After pilot study and item selection analysis, the final and formal medication literacy scale for hypertensive patients has been accomplished, and 4 domains with 37 items were identified. Knowledge domain (K) includes 9 items, attitude domain (A) involves 8 items, skill domain (S) has 7 items, and practice domain (P) has 13 items.

### Validity Analysis

#### Content and Face Validity

Based on the feedback of the two-round expert consultation, two items in the attitude domain were removed. The results of the expert consultation also showed that the individual authority coefficient of each expert ranged from 0.79 to 0.97, the integrated authority coefficient of all experts was 0.92, indicating that the appraisements and suggestions generated from this expert panel were relatively authorized and can be trusted. The active coefficient for all experts in two rounds of expert consultation was 1, which means that each expert in this consultation stage was willing and active to participate to appraise on this scale.

Furthermore, the K^*^ values (Polit et al., 2007) of each item were all > 0.74, indicating a good adjusted chance agreement on item appraisements for experts.

The I-CVI (item-level content validity index) of each item in this scale ranged from 0.833 to 1.000, which were all > 0.78 (Lynn, 1986); the S-CVI/Ave (scale-level content validity index) for the knowledge domain of the scale was 0.962, S-CVI for the attitude domain was 0.979, S-CVI for the practice domain was 0.961, S-CVI for the skill domain was 0.976; the S-CVI for the overall scale was 0.968. The S-CVI for domains and the overall scale were all >0.90 (Waltz et al., 2005). Therefore, a good face and content validity for items in this scale were identified.

#### Exploratory Factor Analysis

Exploratory factor analysis (EFA) was conducted. Principle component analysis with Varimax rotation was employed to analyze the construct and factor structure of this scale and of each domain. Two hundred and fifty-seven collected questionnaires were randomly abstracted from total questionnaires of 637 to conduct the exploratory factor analysis for the scale. Sampling adequacy and factorability of a correlation matrix were evaluated. Kaiser–Mayer–Olkin (KMO) test for the overall scale and its four domains indicated that the sample size for this scale were adequate with values of 0.715 for the scale, and of 0.765, 0.766, 0.713, and 0.808 for its domains, i.e., KMO > 0.7 (Cronbach and Meehl, 1955; Şencan, 2005), Bartlett’s test was significant beyond 0.001. The findings indicated the suitability of the correlation matrix to draw factors.

The results of EFA showed that four components with eigenvalues > 1.0 were identified (Cronbach and Meehl, 1955). Items with factor loadings greater than 0.4 on the same component, as well as non-salient loadings less than 0.4 on other components, were considered to contribute to a component (Zwick and Velicer, 1986; Toll et al., 2007).

According to factor loadings for 37 items in the present study, 37 items contributed to four components respectively. However, in our results, the factor loadings of 37 items for its belonging components were not so good. Suboptimal item factor loadings for anticipated domains for 37 items in this sample were generated, the total explained variance of four components for the overall scale in our sample was 51.42% (**Table 2**). Therefore, we decided to do a further sub-factor extraction from domains. Then, several sub-domains (sub-factors) were extracted from each of the four identified domains. A total of 11 sub-factors for domains were yielded. We found that the total explained variances for domains were 56.111–64.419%, which is fairly good. Therefore, four components of the overall scale and eleven sub-factors for domains were identified. Domain 1 (knowledge) contained 9 items, domain 2 (attitude) contained 8 items, domain 3 (skill) contained 7 items, and domain 4 (practice) contained 13 items. Specifically, in the knowledge domain, three sub-factors with eigenvalues of >1 explained the 64.419% of the total variation. Four items of K6–K9 loaded on sub-factor one measured knowledge for antihypertensive medication; sub-factor two contained three items of K1–K3 that measured patients’ knowledge for hypertension disease; sub-factor three included two items of K4–K5 that measured knowledge for hypertension treatment (**Table 3**).

**Table 2:** Exploratory factor analysis on medication literacy (ML) scale for hypertensive patients (n=257).

	Factors			
	1	2	3	4
K1	**.511**			
K2	**.478**			
K3	**.489**			
K4	**.518**			
K5	**.413**			
K6	**.554**			
K7	**.456**			
K8	**.631**			
K9	**.601**			
A1		**.481**		
A2		**.413**		
A3		**.437**		
A4		**.496**		
A5		**.460**		
A6		**.468**		
A7		**.428**		
A8		**.470**		
P1			**.416**	
P2			**.587**	
P3			**.402**	
P4			**.532**	
P5			**.454**	
P6			**.486**	
P7.1			**.422**	
P7.2			**.320**	
P7.3			**.463**	
P7.4			**.465**	
P8			**.591**	
P9.1			**.450**	
P9.2			**.454**	
S1				**.543**
S2				**.336**
S3				**.505**
S4				**.451**
S5				**.471**
S6				**.499**
S7				**.489**
Eigenvalues	1.545	1.769	1.913	1.063
Explained Variations (%)	12.324	11.541	15.342	12.213

K, knowledge domain; A, attitude domain; S, skill domain; P, practice domain; KMO measure of sampling adequacy value=0.715, Bartlett’s test: χ2 (chi square test value) =651.992; df (degree of freedom) =248; P=0.000.

**Table 3:** Exploratory factor analysis on knowledge domain of medication literacy (ML) scale for hypertensive patients (n=257).

Items	Sub-factors (sub-domains)		
	1	2	3
K1		0.688	
K2		0.867	
K3		0.813	
K4			0.614
K5			0.866
K6	0.776		
K7	0.747		
K8	0.761		
K9	0.795		
Eigenvalues	2.473	2.008	1.317
Explained variations (%)	27.481	22.306	14.631
Factors designation	Sub-domain 1	Sub-domain 2	Sub-domain 3

ML, medication literacy; K, knowledge domain of ML; KMO (Kaiser-Meyer-Olkin) measure of sampling adequacy value=0.765, Bartlett’s test:χ2 (chi square test value) =627.670; df (degree of freedom) =36; P=0.000.

Sub-domain 1: knowledge for antihypertensive medication.

Sub-domain 2: knowledge for hypertension disease.

Sub-domain 3: knowledge for hypertension treatment.

Two common sub-factors with eigenvalues of >1 were extracted from attitude domain, explaining 60.914% of the total variation of this domain. There were five items of A4–A8 loaded on sub-factor one measuring patients’ attitude to taking antihypertensive medication; sub-factor two included three items of A1–A3 that represented patients’ attitude and recognition to the severity of hypertension disease, as well as the necessity to be treated and controlled (**Table 4**).

**Table 4:** Exploratory factor analysis on attitude domain of medication literacy (ML) scale for hypertensive patients (n=257).

Items	Sub-factors (sub-domains)	
	1	2
A1		0.690
A2		0.826
A3		0.808
A4	0.763	
A5	0.776	
A6	0.767	
A7	0.727	
A8	0.785	
Eigenvalues	2.971	1.902
Explained variations (%)	37.134	23.780
Factors designation	Sub-domain 1	Sub-domain 2

A, attitude domain of ML; KMO measure of sampling adequacy value=0.766, Bartlett’s test:χ2 (chi square test value) =723.104; df (degree of freedom) =28; P=0.000.

Sub-domain 1: patients’ attitude to taking antihypertensive medication.

Sub-domain 2: patients’ attitude and recognition to the severity of hypertension disease, as well as the necessity to be treated and controlled.

There were four common sub-factors with eigenvalues of >1 being extracted from practice domain and they explained 59.474% of the total variation of this domain. Four items of P7.1–P7.4 loaded on sub-factor one representing antihypertensive medication taking adherence behavior; sub-factor two had three items of P4–P6 that measured medication use decision making behavior; three items of P8, P9.1, P9.2 loaded on sub-factor three representing patients’ blood pressure self-monitoring and surveillance; sub-factor four contained three items of P1–P3 that represented antihypertensive medication information-seeking and dissemination behavior (**Table 5**).

**Table 5:** Exploratory factor analysis on practice domain of medication literacy (ML) scale for hypertensive patients (n=257).

Items	Sub-factors (sub-domains)			
	1	2	3	4
P1				0.489
P2				0.652
P3				0.671
P4		0.789		
P5		0.722		
P6		0.713		
P7.1	0.766			
P7.2	0.803			
P7.3	0.766			
P7.4	0.757			
P8			0.476	
P9.1			0.659	
P9.2			0.654	
Eigenvalues	2.512	1.988	1.762	1.469
Explained Variations (%)	19.327	15.294	13.553	11.300
Factors designation	Sub-domain 1	Sub-domain 2	Sub-domain 3	Sub-domain 4

P, practice domain of ML; KMO measure of sampling adequacy value=0.713, Bartlett’s test: χ2 (chi square test value) =874.831; df (degree of freedom) =78; P=0.000.

Sub-domain 1: adherence to antihypertensive medication taking behavior.

Sub-domain 2: medication use decision making behavior.

Sub-domain 3: blood pressure self-monitoring and surveillance.

Sub-domain 4: antihypertensive medication information-seeking and dissemination behavior.

In addition, two common sub-factors with eigenvalues of >1 were extracted from skill domain, explaining 56.111% of the total variation of this domain. Four items of S4–S7 loaded on sub-factor one measuring patients’ ability to read and comprehend the prescription and medication instruction; sub-factor two contained three items of S1–S3 that measured patients’ ability to do numeric calculation correctly for administered dosage of antihypertensive medication, time for medication taking, as well as time for prescription refill (**Table 6**).

**Table 6:** Exploratory factor analysis on skill domain of medication literacy (ML) scale for hypertensive patients (n=257).

Items	Sub-factors (sub-domains)	
	1	2
S1		0.704
S2		0.750
S3		0.643
S4	0.600	
S5	0.789	
S6	0.739	
S7	0.821	
Eigenvalues	2.275	1.653
Explained variations (%)	32.503	23.608
Factors designation	Sub-domain 1	Sub-domain 2

S, skill domain of ML; KMO measure of sampling adequacy value=0.808, Bartlett’s test:χ2 (chi square test value) =373.837; df (degree of freedom) =21; P=0.000.

Sub-domain 1: patients’ ability to read and comprehend the prescription and medication instruction.

Sub-domain 2: patients’ ability to do numeric calculation correctly for administered dosage of antihypertensive medication, time for medication taking, as well as time for prescription refill.

In this stage, four domains along with 11 sub-factors in domains have been explored. This result was complied with the concept of medication literacy with four core elements of knowledge, attitude, skill, and practice. Subsequently, this four-domain model was then confirmed in the rest sample by conducting a confirmatory factor analysis (CFA).

#### Confirmatory Factor Analysis

**Figure 1:**
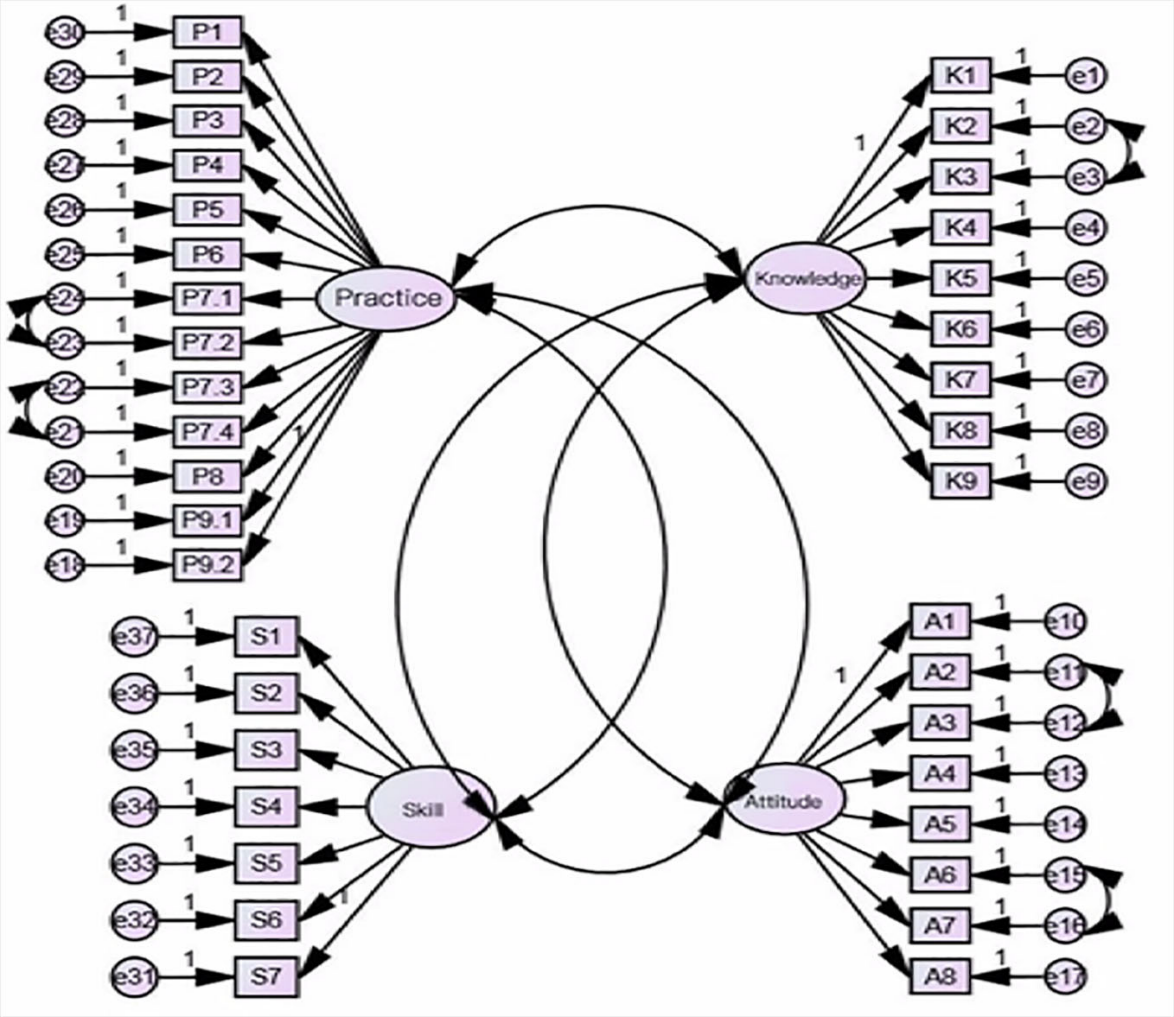
Structure equation modeling of four-domain with 11 sub-factors for medication literacy scale. P1-P9.2: items for practice domain; K1–K9: items for knowledge domains; S1–S7: items for skill domain; A1–A8: items for attitude domain.

Out of the 637 collected questionnaires we used three hundred and eighty questionnaires to test the 4-domain model of the scale. Fit indices were calculated. The values obtained for fit indices for the overall scale in CFA were as follows: IFI = 0.746, i.e., near to 0.9. The values for RMSEA and RMR was 0.066 and 0.012, respectively, i.e., less than 0.07. The values for GFI, AGFI were 0.804, 0.777, i.e., near to 0.9. Values for PCFI and PNFI were 0.689 and 0.599, i.e., > 0.50. In our results, the value of χ^2^/df was 2.629, i.e., < 3. Therefore, all indices except IFI, GFI, and AGFI, meet the fitting requirements, demonstrating that the four-domain model had acceptable factorial validity in current sample (**Table 7**). The structure equation modeling was showed in **Figure 1**. For convergent validity and discriminant validity, in our results for this scale, the standardized factor loadings of this scale were reported larger than 0.5 and statistically significant (Hair et al., 2010). Therefore, the convergent validity of this scale was satisfied; additionally, the average variance and squared correlation coefficients among domains were also calculated and the results showed that the average variance values were greater than their respective squared correlation coefficients (Hair et al., 2010). This indicates that the discriminant validity was satisfied.

**Table 7:** The results of fitting indices of confirmatory factor analysis of four-domain model of medication literacy assessment scale for hypertensive patients (n=380).

Parameters	Four-domain model
χ^2^/df	2.629 < 3
GFI	0.804 near to 0.90
AGFI	0.777 near to 0.90
RMR	0.012 < 0.05
IFI	0.746 near to 0.90
RMSEA	0.066 < 0.08
PCFI	0.689 > 0.05
PNFI	0.599 > 0.05

### Reliability Analysis

The Cronbach’s α coefficient, split-half reliability, and test-retest reliability coefficient of the overall scale and of each domain were measured. The Cronbach’s α coefficient of the overall scale for 37 items was 0.849. i.e., > 0.7. All items were positively correlated with its domain and the overall scale. The Cronbach’s α coefficients for each domain ranged from 0.744 to 0.783. i.e., > 0.7. Therefore, good internal consistency reliability for the overall scale and for each domain were established. The Spearman-Brown split-half correlation coefficient for the overall scale was 0.893, for each domain, it ranged from 0.793 to 0.872, indicating high internal consistencies between the halves across scale and its domains. The test-retest Pearson’s correlation coefficient for the overall scale was 0.968, for each domain of the scale, it ranged from 0.880 to 0.959 (P-value < 0.01), confirming the consistency of the scale and its subscales (domains) over time (**Table 8**). In addition, the Pearson’s correlation coefficients were used to identify the direction and degree of association between the scale and its domains. There were no exact boundaries; however, a correlation coefficient of 0.50 or lower showed weak, between 0.50 and 0.70 corresponded to an average, and a correlation of >0.70 indicated a strong association (Durmuş et al., 2011). In this study, the Pearson’s correlation coefficients between each domain and the overall scale ranged from 0.530 to 0.799 (*P* < 0.01), indicating an average to strong association between the scale and its domains was identified. The Pearson’s correlation coefficients among domains ranged from 0.157 to 0.439 (*P* < 0.01) (**Table 9**), indicating a weak association among domains in this scale was confirmed. Therefore, good reliability and acceptable validity of this scale were established.

**Table 8:** The reliability coefficients of the total scale and among each dimension of medication literacy assessment scale for hypertensive patients (n=637).

Domains	Items	Cronbach’s α coefficient	Spearman-Brown split-half reliability	Test-retest reliability
K	9	0.754	0.816	0.958
A	8	0.783	0.872	0.959
P	13	0.744	0.809	0.928
S	7	0.763	0.793	0.880
ML	37	0.849	0.893	0.968

**Table 9:** Latent correlations among domains and the overall scale for hypertensive patients (n=637).

	ML	K	A	P	S
ML	1				
K	0.799^**^	1			
A	0.530^**^	0.283^**^	1		
P	0.746^**^	0.439^**^	0.334^**^	1	
S	0.653^**^	0.370^**^	0.157^**^	0.216^**^	1

**Statistically significant correlation with each other at level of 0.01 (bilateral). ML, medication literacy; K, knowledge domain; A, attitude domain; P, practice domain; S, skill domain.

## Discussion

This is the first study to develop a self-reporting medication literacy scale specific for hypertensive patients (MLSHP), which is also aimed at exploring the psychometric properties of MLSHP in Chinese hypertensive patients. Though, there were several existing medication literacy scales for general population. For example, Medication Literacy Assessment Scale in Spanish and English (MedLitRxSE) (Sauceda et al., 2012), which included 14 items and was unidimensional, and it was developed mainly aiming to evaluate patients’ document literacy and numeracy; Chinese Medication Literacy Measure (ChMLM) with 17 items in four sections mainly aimed to assess individual’s ability to interpret medication-related vocabulary, to read and comprehend prescription and non-prescription drug instructions, as well as to evaluate a drug advertisement (Yeh et al., 2017), however, the factor loadings of items for its anticipated four components and the item-total-correlations were not so good; Medication Literacy Assessment Questionnaire with 30 items in five categories aimed at assessing patients’ ability to read and understand medication instruction sheets (Horvat et al., 2017), but only content validity was established in this questionnaire. In addition, pharmacotherapy literacy questionnaire for parents of pre-school children was designed based on defined domains of pharmacotherapy literacy on knowledge, understanding, numerical skills, and access to medicines-related information (Ubavić et al., 2018b), but the KR-20 was only 0.542 and split-half reliability coefficient was 0.436, which was suboptimal for this questionnaire. In addition, the construct validity was not applied for this questionnaire. In particular, among these existing scales measuring medication literacy or pharmacotherapy literacy, there were not consistent defined domains for medication literacy, and they were mainly for general population. Therefore, we decided to operationalize the concept of medication literacy based on an international consensus of medication literacy, and to develop this present scale specific for hypertensive patients.

In the EFA of the present study, we identified four domains of knowledge, attitude, skill, and practice as anticipated, however, the factor loadings of items for the whole scale in each factor (domain) were not optimal. Therefore, we continued to do a sub-factor extraction from four domains. It turned out that 11 sub-factors have explained the variances for its belonging domains in a good way. The 11 sub-factors were exactly consistent with the indicator system of medication literacy for hypertensive patients we initially established. Firstly, the indicator system was generated based on the operationalized four core elements of medication literacy, the initial item pool was subsequently formulated based on the indicator system.

CFA was conducted to test the goodness-of-fit for the identified four-domain model. All indices except IFI, GFI, and AGFI meet the fitting requirements. GFI, AGFI, IFI were 0.804, 0.777, 0.746, which were all near to 0.9. we considered that the four-domain model had acceptable factorial validity in current sample but the model fit still needs to be further confirmed.

In addition, the standardized factor loadings and the latent correlations among factors (domains) testified that the four domains are not only relative independent but also correlated with each other, which was consistent with the anticipated relationship among four operationalized elements in the present study. Due to four domains of this scale were correlated with one another to some extent, we suggested that future study could also focus on conducting a path analysis among four operationalized elements of knowledge, attitude, skill, and practice, in order to figure out a specific interaction path among four elements and find the mechanism that how these four operationalized elements correlate or interact with each other.

In the present study, content and face validity as well as construct validity were measured. Results showed that good content and face validity of this newly developed medication literacy scale for hypertensive patients was satisfactory, indicating that the items of this scale was appropriate to its domains as well as to its whole scale and consensus of experts on items were achieved.

In this study, the internal consistency of this scale was measured. The Cronbach’s α coefficient of the overall scale for 37 items was 0.849. This was higher than the Cronbach’s alpha value reported by ChMLM scale among general population in Taiwan (Yeh et al., 2017), i.e., 0.72. Furthermore, the split-half reliability correlation coefficient of this studied scale (0.893) was higher than that of a 14-item English and Spanish MedLitRxSE tool for general population (Sauceda et al., 2012), i.e., (English: KR-20 = 0.81; Spanish: KR-20 = 0.77). Therefore, the newly developed scale was highly reliable to measure what it was supposed to test as it was designed.

In addition, this scale demonstrated a high acceptability among hypertensive patients with a response rate of 96.6 and 98%. Therefore, this newly developed scale is easy to use and fill in, which is pragmatic and applicable in assessing hypertensive patients’ medication literacy in China.

The strengths established in this study: this is a new and the first specific patient-reported outcomes instrument for hypertensive patients; the developed scale in this study is proved with high patient acceptability, a rigorous and scientific procedure of measurement purification. In addition, greatly satisfied reliability, content validity, convergent and discriminate validity of this newly developed scale was confirmed.

Limitations: the data collected was self-reported by patients, there is a key problem about the subjectivity of patients’ response to this medication literacy scale. Therefore, the quality and accuracy of the data collected in this study might be compromised. In addition, the construct validity for this medication literacy scale was not satisfactory enough according to the EFA results of the whole scale and the suboptimal indices of GFI, AGFI, and IFI in the CFA, i.e., < 0.9. Therefore, further study on this scale is still necessary in order to re-confirm the construct validity of the overall scale. The validation of this newly developed medication literacy scale for hypertensive patients in other sample of population in China is still needed, may be with some minor adaptations for items. Besides, English translation and validation and adaptation is also required for its international utilization.

## Conclusions

A newly self-reporting medication literacy scale for hypertensive patients was developed in Chinese language. The measurement property of this scale has been established, in which good reliability and acceptable validity were confirmed, suggesting its appropriateness and applicability to measure medication literacy level for Chinese hypertensive patients. Future study will be focused mainly on three aspects: first, further validation and adaptation of this scale, mainly on construct and model fit validation, is still needed; second, English translation is needed, so that this scale application can be further validated and adapted worldwide; finally, large-scale investigation of hypertensive patients’ medication literacy in China based on this scale is needed, so associated factors of hypertensive patients’ medication level could be found.

## Data Availability Statement

All datasets generated for this study are included in the article/**Supplementary Material**.

## Ethics Statement

The studies involving human participants were reviewed and approved by the Ethics Committee of the Third Xiangya Hospital. The patients/participants provided their written informed consent to participate in this study.

## Author Contributions

AL was in charge of the whole design. ZZ and SS were in charge of the paper writing and data collection. YD, ZS, FZ and SD made contributions to the statistical data analysis and data collection.

## Funding

This study was supported and funded by the Youth Program of National Natural Science Foundation of China (Project Grant Number: 71603290).

## Conflict of Interest

The authors declare that the research was conducted in the absence of any commercial or financial relationships that could be construed as a potential conflict of interest.
